# *In vitro* activity of gepotidacin against baseline *Neisseria gonorrhoeae* phenotypes and genotypes recovered from a clinical trial for uncomplicated urogenital gonorrhea (EAGLE-1)

**DOI:** 10.1128/aac.00752-26

**Published:** 2026-10-07

**Authors:** Charles Jakielaszek, William Flight, Dan Lythgoe, Caroline Perry, Nicole E. Scangarella-Oman

**Affiliations:** 1 Infectious Diseases Research, GSK 73111 , Collegeville, Pennsylvania, USA; 2 Infectious Diseases Research, GSK 1929 , London, United Kingdom; 3 Biostatistics, GSK, Stevenage, United Kingdom; University of Pennsylvania Perelman School of Medicine, Philadelphia, Pennsylvania, USA

**Keywords:** antimicrobial, antimicrobial activity, clinical trial, EAGLE-1, *in vitro*, gepotidacin, genotype, *Neisseria gonorrhoeae*, phenotype, uncomplicated urogenital gonorrhea

## Abstract

There is an urgent need for novel antimicrobials to treat multidrug-resistant strains of *Neisseria gonorrhoeae*. In a phase III trial (EAGLE-1), gepotidacin, a first-in-class triazaacenaphthylene antibacterial, demonstrated non-inferiority to ceftriaxone plus azithromycin as measured by microbiological success for the treatment of uncomplicated urogenital gonorrhea. Here, we assessed the *in vitro* activity of gepotidacin against key *N. gonorrhoeae* drug-resistant phenotypes and genotypes from EAGLE-1. There were 408 baseline urogenital isolates recovered from 406 patients during the EAGLE-1 study. Agar dilution minimum inhibitory concentration (MIC) susceptibility testing was performed according to Clinical and Laboratory Standards Institute (CLSI) guidelines, and all isolates were molecularly characterized. All of the isolates were susceptible to ceftriaxone. All isolates had gepotidacin MICs of ≤2 µg/mL, with values ranging from ≤0.06 to 2 µg/mL; MIC_50_, 0.25 µg/mL, and MIC_90_, 0.5 µg/mL. As defined by the Food and Drug Administration (FDA) breakpoints and interpretive criteria (MIC breakpoints of ≤1 µg/mL [susceptible]; 2 µg/mL [intermediate]; and ≥4 µg/mL [resistant]), 99% of the study isolates were susceptible to gepotidacin, 1% were intermediate, and no isolates were resistant. Susceptibility percentages remained high across resistance phenotypes and genotypes. These findings support the *in vitro* activity of gepotidacin against the *N. gonorrhoeae* isolates, including drug-resistant phenotypes and genotypes, recovered in the EAGLE-1 clinical study.

**CLINICAL TRIALS:**

This study is registered with ClinicalTrials.gov as NCT04010539.

## INTRODUCTION

Gonorrhea is a globally prevalent bacterial infection and remains one of the most common sexually transmitted infections worldwide, with an estimated 82 million new cases in adolescents and adults in 2020 (1–3). The infection, which is often asymptomatic, is caused by *Neisseria gonorrhoeae*, a bacterium that primarily infects urogenital sites but may also be detected at extragenital locations such as the pharynx and rectum (2–4). When left untreated, gonorrhea can lead to serious and potentially long-term complications in both men and women (1, 3, 4). The treatment and management of this infection, for which there is currently no approved gonorrhea-specific vaccine, represents a significant and increasing global burden on healthcare systems and economies. Drug-resistant *N. gonorrhoeae* has been identified by international and national organizations as a major and urgent public health threat (4–7).

Antibiotics are the mainstay treatment for gonorrhea, and current recommendations center on parenteral ceftriaxone for first-line treatment (8–10). The effective pharmacological treatment of gonorrhea has become increasingly challenging due to the emergence and spread of antimicrobial resistance and multidrug-resistant strains of *N. gonorrhoeae* (7, 8, 10–13). Among existing strains, *N. gonorrhoeae* has developed or acquired resistance to almost all antibacterials used to treat gonorrhea, including penicillins, tetracyclines, sulfonamides, macrolides, and quinolones (14). The first global treatment failure of dual therapy with ceftriaxone plus azithromycin was reported nearly 10 years ago, and multidrug-resistant *N. gonorrhoeae* strains have emerged and are spreading, including those resistant to both ceftriaxone and azithromycin (10, 15). Concerns over drug resistance have resulted in ceftriaxone monotherapy being the only remaining first-line treatment for gonorrhea recommended by the World Health Organization and Centers for Disease Control and Prevention (CDC) in the United States (US) (7, 14).

There is an urgent unmet need for novel antimicrobials to treat gonorrhea, especially the treatment of drug-resistant strains. Moreover, the development of new oral treatment options offers potential advantages over parenteral administration. The approvals of the oral antibacterial agents gepotidacin and zoliflodacin represent important advances in expanding treatment options for uncomplicated gonorrhea (16, 17), particularly in the context of increasing antimicrobial resistance (14).

Gepotidacin is an oral, first-in-class, bactericidal, triazaacenaphthylene antibacterial that inhibits bacterial DNA replication by a distinct binding site (on GyrA and ParC) and has a unique mechanism of action (18–20). This provides well-balanced inhibition (for most target pathogens including *N. gonorrhoeae*) of two type II topoisomerase enzymes (DNA gyrase and topoisomerase IV) (20–22), such that a gepotidacin target-specific mutation in one enzyme only does not substantially affect gepotidacin susceptibility (20). Combined, these properties give gepotidacin activity against a range of target uropathogens, such as *Escherichia coli* and *Staphylococcus saprophyticus,* as well as *N. gonorrhoeae*, including strains resistant to current antibacterials (23–25).

EAGLE-1 (NCT04010539) was a phase III, open-label, active-comparator, non-inferiority trial evaluating the efficacy and safety of oral gepotidacin for the treatment of uncomplicated urogenital gonorrhea (26). The study used an optimized dosing regimen of two 3,000 mg doses given 10–12 h apart, based on findings from a phase II study (27). In addition, the study assessed antimicrobial resistance prevalence in *N. gonorrhoeae* isolates using both phenotypic susceptibility profiles and genotypic markers (26). As previously reported, baseline *N. gonorrhoeae* isolates were characterized by resistance to ciprofloxacin (54%), tetracycline (25%), and penicillin (17%), with quinolone resistance-determining region (QRDR) mutations identified in 59% of isolates (26). Gepotidacin was non-inferior to ceftriaxone plus azithromycin for microbiological success (bacterial eradication of urogenital *N. gonorrhoeae*) at the test-of-cure visit (days 4–8 post-treatment), achieving rates of 92.6% (95% confidence interval [CI]: 88.0–95.8%) compared with 91.2% (95% CI: 86.4–94.7%) for ceftriaxone/azithromycin (adjusted treatment difference: −0.1% [95% CI: −5.6% to 5.5%]) (26). In the microbiologically evaluable population (i.e., participants in the microbiological intent-to-treat [micro-ITT] population who followed important components of the trial protocol), the microbiological success rate was 100% in both treatment groups (26). There was no bacterial persistence of *N. gonorrhoeae* at the urogenital site in either treatment group, and gepotidacin maintained efficacy against *N. gonorrhoeae* isolates resistant or nonsusceptible to existing antibacterials (26). Gepotidacin had an acceptable safety and tolerability profile, consistent with earlier phase studies (26, 28).

In this analysis, we evaluated the activity of gepotidacin by assessing minimum inhibitory concentration (MIC) summary statistics including frequency distributions and the proportion of isolates susceptible to gepotidacin based on the approved Food and Drug Administration (FDA) susceptibility test interpretive criteria (STIC), both overall and against drug-resistant *N. gonorrhoeae* isolates recovered in EAGLE-1.

## MATERIALS AND METHODS

### Study design

The study design and participant eligibility criteria have been described previously (26). Briefly, EAGLE-1 was a phase III, multicenter study conducted from October 2019 to October 2023 evaluating oral gepotidacin (3,000 mg administered twice, 10–12 h apart) in adolescents and adults with uncomplicated urogenital gonorrhea with or without rectal and/or pharyngeal *N. gonorrhoeae* infection. Information on sex and sexual orientation was self-reported by participants (26).

The study was conducted at 49 centers across Spain, Germany, Australia, the United Kingdom (UK), the United States, and Mexico, in accordance with the principles of the Declaration of Helsinki and the International Council for Harmonisation Guidelines for Good Clinical Practice. The study protocol was approved by relevant research ethics committees and institutional review boards, as appropriate for participating study centers. All participants provided written informed consent prior to study entry.

### Microbiological assessments

Bacteriological culture for *N. gonorrhoeae* was performed at the baseline and test-of-cure (days 4–8 post-treatment) visits using urogenital swab specimens collected from all participants. Rectal and pharyngeal specimens were also collected from participants who provided consent. Culture was performed at follow-up (days 14–21) from the pharyngeal body site only if both the baseline and test-of-cure pharyngeal nucleic acid amplification test were positive for *N. gonorrhoeae*.

Microbiological assessments for *N. gonorrhoeae*, including primary culture and presumptive identification of *N. gonorrhoeae*, were performed at local laboratories, with confirmation of identification and susceptibility testing conducted at central laboratories (PPD Laboratory Services Central Lab, Highland Heights, KY, USA, and JMI Laboratories; now part of Element; North Liberty, IA, USA). Minimum inhibitory concentrations were determined by agar dilution according to Clinical and Laboratory Standards Institute (CLSI) guidelines (29, 30). Antibacterials tested included gepotidacin, azithromycin, cefixime, ceftriaxone, ciprofloxacin, gentamicin, penicillin, spectinomycin, and tetracycline.

Molecular characterization using whole-genome sequencing was performed at a central laboratory (JMI Laboratories). Total genomic DNA was extracted from swab specimens using KingFisher Flex Magnetic Particle Processors (Thermo Scientific, Cleveland, OH, USA) and was used to construct DNA libraries using the Nextera XT library construction protocol, according to the manufacturer’s instructions (Illumina, San Diego, CA, USA). Sequencing was performed on a MiSeq Sequencer (Illumina). Screening was undertaken for mutations including the quinolone resistance-determining region (QRDR) and Mtr region, and for multi-locus sequence typing (MLST) and *N. gonorrhoeae* multi-antigen sequence typing (NG-MAST).

The current study evaluated gepotidacin MIC summary data, including frequency distributions and susceptibility to gepotidacin, overall and by key phenotype and genotypes.

### Statistical analysis

The analyses presented here were conducted on *N. gonorrhoeae* isolates obtained from the micro-ITT population, which was the primary analysis population, as described previously (26). The micro-ITT population included all randomized participants who received at least one dose of study drug and had a confirmed baseline urogenital isolate of *N. gonorrhoeae* that was susceptible to ceftriaxone based on CLSI breakpoints (26). Isolates from the rectal and pharyngeal body sites included in this analysis were all obtained from participants in the micro-ITT population.

The prevalence of the different drug-resistance phenotypes and genotypes is reported as the percentage of isolates exhibiting each characteristic among all isolates within the indicated treatment group or subgroups. Prevalence percentages are presented for phenotypes and genotypes with ≥10 results.

Gepotidacin MIC frequency distributions are presented as the number of isolates inhibited and cumulative percentage. Gepotidacin susceptibility is based on FDA STIC breakpoints (31), as CLSI breakpoints were not available at the time of this analysis. Antimicrobial susceptibility interpretations for comparator antimicrobial agents are based on CLSI M100 guidelines (30).

## RESULTS

### Demographic and clinical characteristics for the micro-ITT population

Of the 406 participants in the micro-ITT population, most were male (372, 91.6%) and the most common sex and sexual orientation category was men who have sex with men (MSM, 290, 71.4%), followed by men who have sex with women (MSW, 82, 20.2%) and female (34, 8.4%). The age of participants ranged from 17 to 64 years, with a mean age of 33.1 years as previously reported (26).

### Prevalence of *N. gonorrhoeae* phenotypes and genotypes

A total of 408 baseline urogenital *N. gonorrhoeae* isolates from the 406 participants in the micro-ITT population were available for analysis. Two participants had two baseline urogenital isolates. A total of 41 baseline rectal *N. gonorrhoeae* isolates (micro-ITT rectal population) and 35 baseline pharyngeal *N. gonorrhoeae* isolates (micro-ITT pharyngeal population) were available for analysis.

The prevalence of *N. gonorrhoeae* antimicrobial-resistance/nonsusceptibility phenotypes and genotypes in the overall micro-ITT population was previously reported (26).

### *In vitro* antimicrobial activity of gepotidacin and comparators

Gepotidacin was active *in vitro* against the 408 baseline urogenital *N. gonorrhoeae* isolates tested, with MIC values ranging from ≤0.06 to 2 µg/mL and MIC_50_ and MIC_90_ values of 0.25 and 0.5 µg/mL, respectively. Susceptibility of urogenital *N. gonorrhoeae* isolates to gepotidacin and the comparators is shown in Table 1. The percentage of isolates susceptible to gepotidacin was 99% (404/408), with 1% (4/408) of isolates classified as intermediate according to the FDA STIC breakpoints. As per the CLSI M100 criteria, all isolates (408/408 [100%]) were susceptible to ceftriaxone and cefixime, and 407/408 isolates (99.8%) were susceptible to spectinomycin. In contrast, susceptibility was substantially lower for penicillin (10/408 [2.5%]), tetracycline (84/408 [20.6%]), and ciprofloxacin (178/408 [43.6%]). Azithromycin susceptibility was observed in 371/408 (90.9%) isolates, with 37/408 (9.1%) categorized as nonsusceptible.

**TABLE 1:** Antimicrobial activity of gepotidacin and comparator antimicrobial agents against urogenital *N. gonorrhoeae* isolates at baseline (*N* = 408 isolates) (micro-ITT population)^*a*^

Antimicrobial agent	MIC (µg/mL)			Interpretation (*n* [%])^*b*^			
	MIC_50_^*c*^	MIC_90_^*d*^	Range	Susceptible	Intermediate	Resistant	Nonsusceptible
Gepotidacin	0.25	0.5	≤0.06 to 2	404 (99.0)	4 (1.0)	0	–
Ciprofloxacin	1	8	0.002 to 32	178 (43.6)	11 (2.7)	219 (53.7)	–
Ceftriaxone	0.008	0.015	≤0.002 to 0.12	408 (100)	–	–	0
Cefixime	0.015	0.06	≤0.002 to 0.25	408 (100)	–	–	0
Azithromycin	0.25	1	≤0.03 to >256	371 (90.9)	–	–	37 (9.1)
Penicillin	0.5	4	≤0.06 to 128	10 (2.5)	329 (80.6)	69 (16.9)	–
Tetracycline	0.5	32	≤0.12 to 64	84 (20.6)	222 (54.4)	102 (25.0)	–
Spectinomycin	32	32	≤4 to 64	407 (99.8)	1 (0.2)	0	–
Gentamicin	8	8	≤1 to 16	–	–	–	–

^*a*^ CLSI, Clinical and Laboratory Standards Institute; FDA, Food and Drug Administration; MIC, minimum inhibitory concentration; micro-ITT, microbiological intent-to-treat; *N. gonorrhoeae*, *Neisseria gonorrhoeae*.

^*b*^ Percentages for interpretations are calculated from *n/N* where *N* is the total number of *N. gonorrhoeae* isolates (*N *= 408). Gepotidacin interpretations are based on FDA susceptibility test interpretive criteria (susceptible ≤1 µg/mL; intermediate = 2 µg/mL; and resistant ≥4 µg/mL). Susceptibility interpretations for comparator antimicrobial agents are based on CLSI M100 guidelines. – indicates data are not available.

^*c*^ MIC_50_ represents the minimum concentration at which 50% of tested bacterial isolates are inhibited in growth.

^*d*^ MIC_90_ represents the minimum concentration at which 90% of tested bacterial isolates are inhibited in growth.

### *In vitro* activity of gepotidacin across drug-resistant phenotypes and genotypes

The *in vitro* antimicrobial activity of gepotidacin was evaluated against *N. gonorrhoeae* isolates according to drug-resistant/nonsusceptible phenotype and genotype subgroups (Table 2; Tables S1 and S2).

**TABLE 2:** Gepotidacin MIC frequency distribution and summary statistics against urogenital *N. gonorrhoeae* isolates by phenotype and genotype at baseline (*N* = 408 isolates) (micro-ITT population)^*a*^

Phenotype or genotype subgroup^*b*^	No. of isolates	No. of isolates (cumulative %) inhibited with gepotidacin MIC (µg/mL)^*c*^						MIC_50_ (µg/mL)^*d*^	MIC_90_ (µg/mL)^*e*^	Interpretation (*N* [%])^*f*^		
		≤0.06	0.12	0.25	0.5	1	2			Susceptible	Intermediate	Resistant
All isolates	408	40 (9.8)	98 (33.8)	170 (75.5)	70 (92.6)	26 (99.0)	4 (100)	0.25	0.5	404 (99.0)	4 (1.0)	0
Phenotypes, *n* (%)												
Azithromycin-nonsusceptible	37	2 (5.4)	3 (13.5)	8 (35.1)	20 (89.2)	3 (97.3)	1 (100)	0.5	1	36 (97.3)	1 (2.7)	0
Ciprofloxacin-resistant	219	33 (15.1)	65 (44.7)	68 (75.8)	26 (87.7)	23 (98.2)	4 (100)	0.25	1	215 (98.2)	4 (1.8)	0
Penicillin-resistant	69	2 (2.9)	14 (23.2)	28 (63.8)	13 (82.6)	11 (98.6)	1 (100)	0.25	1	68 (98.6)	1 (1.4)	0
Tetracycline-resistant	102	5 (4.9)	10 (14.7)	39 (52.9)	29 (81.4)	17 (98.0)	2 (100)	0.25	1	100 (98.0)	2 (2.0)	0
Ciprofloxacin- + penicillin- + tetracycline-resistant	47	2 (4.3)	8 (21.3)	13 (48.9)	12 (74.5)	11 (97.9)	1 (100)	0.5	1	46 (97.9)	1 (2.1)	0
Genotypes, *n* (%)												
QRDR mutations	240	34 (14.2)	70 (43.3)	79 (76.3)	30 (88.8)	23 (98.3)	4 (100)	0.25	1	236 (98.3)	4 (1.7)	0
GyrA mutation	236	33 (14.0)	69 (43.2)	79 (76.7)	28 (88.6)	23 (98.3)	4 (100)	0.25	1	232 (98.3)	4 (1.7)	0
GyrA S91F, D95A mutations	213	31 (14.6)	67 (46.0)	70 (78.9)	23 (89.7)	20 (99.1)	2 (100)	0.25	1	211 (99.1)	2 (0.9)	0
ParC mutation	208	33 (15.9)	65 (47.1)	60 (76.0)	23 (87.0)	23 (98.1)	4 (100)	0.25	1	204 (98.1)	4 (1.9)	0
ParC D86N mutation	47	2 (4.3)	2 (8.5)	3 (14.9)	16 (48.9)	20 (91.5)	4 (100)	1	1	43 (91.5)	4 (8.5)	0
ParC S87R mutation	146	30 (20.5)	60 (61.6)	52 (97.3)	4 (100)	0 (100)	0 (100)	0.12	0.25	146 (100.0)	0	0
GyrA S91F, D95A, and ParC D86N mutations	41	2 (4.9)	2 (9.8)	3 (17.1)	13 (48.8)	19 (95.1)	2 (100)	1	1	39 (95.1)	2 (4.9)	0
GyrA S91F, D95A, and ParC S87R mutations	136	28 (20.6)	59 (64.0)	46 (97.8)	3 (100)	0 (100)	0 (100)	0.12	0.25	136 (100.0)	0	0
MtrCDE promoter mutations	140	7 (5.0)	19 (18.6)	54 (57.1)	39 (85.0)	17 (97.1)	4 (100)	0.25	1	136 (97.1)	4 (2.9)	0
MtrC mutation	140	7 (5.0)	19 (18.6)	54 (57.1)	39 (85.0)	17 (97.1)	4 (100)	0.25	1	136 (97.1)	4 (2.9)	0
MtrD mutation	140	7 (5.0)	19 (18.6)	54 (57.1)	39 (85.0)	17 (97.1)	4 (100)	0.25	1	136 (97.1)	4 (2.9)	0
MtrE mutation	140	7 (5.0)	19 (18.6)	54 (57.1)	39 (85.0)	17 (97.1)	4 (100)	0.25	1	136 (97.1)	4 (2.9)	0
MtrR coding mutation	137	7 (5.1)	18 (18.2)	52 (56.2)	39 (84.7)	17 (97.1)	4 (100)	0.25	1	133 (97.1)	4 (2.9)	0
MtrR promoter mutation	46	1 (2.2)	3 (8.7)	9 (28.3)	23 (78.3)	6 (91.3)	4 (100)	0.5	1	42 (91.3)	4 (8.7)	0
23s rRNA mutation	40	2 (5.0)	3 (12.5)	8 (32.5)	20 (82.5)	3 (90.0)	4 (100)	0.5	1	36 (90.0)	4 (10.0)	0

^*a*^ Percentages are calculated from *n/N *where *N* is the number of *N. gonorrhoeae* isolates within the group or subgroup. FDA, Food and Drug Administration; MIC, minimum inhibitory concentration; micro-ITT, microbiological intent-to-treat; *N. gonorrhoeae*, *Neisseria gonorrhoeae*; QRDR, quinolone resistance-determining region; rRNA, ribosomal ribonucleic acid.

^*b*^ Phenotype and genotype categories with *n* ≥ 10 isolates are shown.

^*c*^ The percentage of isolates shown for each gepotidacin MIC concentration was calculated as a cumulative value in a *post hoc* analysis. Gepotidacin MIC values ranged from ≤0.06 to 2 µg/mL; higher MIC categories are thus not shown.

^*d*^ MIC_50_ represents the minimum concentration at which 50% of tested bacterial isolates are inhibited in growth.

^*e*^ MIC_90_ represents the minimum concentration at which 90% of tested bacterial isolates are inhibited in growth.

^*f*^ Gepotidacin interpretations are based on FDA susceptibility test interpretive criteria (susceptible ≤1 µg/mL; intermediate = 2 µg/mL; and resistant ≥4 µg/mL).

#### Antimicrobial-resistant phenotypes

Among baseline urogenital *N. gonorrhoeae* isolates, for all drug-resistant phenotypes, including the combined ciprofloxacin/tetracycline/penicillin-resistance phenotype, gepotidacin MICs ranged from ≤0.06 to 2 µg/mL, MIC_50_ values ranged from 0.25 to 0.5 µg/mL, and MIC_90_ values were 1 µg/mL. Overall, 97.3% of azithromycin-nonsusceptible, 97.9% of combined ciprofloxacin/tetracycline/penicillin-resistant, and 98.6% of penicillin-resistant isolates were susceptible to gepotidacin. All study isolates were inhibited at a gepotidacin MIC of ≤2 µg/mL (Table 2).

Gepotidacin *in vitro* activity against *N. gonorrhoeae* drug-resistant phenotypes from rectal and pharyngeal isolates obtained from the micro-ITT rectal and micro-ITT pharyngeal populations is provided in Tables S1 and S2, respectively. As sampling from these body sites was optional, the number of isolates was small. The only phenotype with ≥10 rectal isolates was the ciprofloxacin-resistant phenotype, for which gepotidacin had an MIC_90_ of 0.5 µg/mL (Table S1). Among pharyngeal isolates, gepotidacin had an MIC_90_ of 1 µg/mL for the ciprofloxacin-resistant and tetracycline-resistant phenotypes (phenotypes with ≥10 pharyngeal isolates) (Table S2).

Gepotidacin susceptibility for antimicrobial-resistant phenotypes was generally similar across urogenital and extragenital sites. A small proportion of urogenital isolates (1.4–2.7%) were categorized as intermediate according to FDA STIC breakpoints. All rectal and pharyngeal isolates obtained from the micro-ITT population were susceptible to gepotidacin.

#### Genotypic subgroups

Against urogenital *N. gonorrhoeae* isolates with QRDR, Mtr, and 23s rRNA mutations, a gepotidacin MIC_90_ of 1 µg/mL was observed for all genotypic subcategories except two (ParC S87R mutations and the GyrA S91F, D95A, and ParC S87R mutation combination), for which the gepotidacin MIC_90_ was 0.25 µg/mL, which is two dilutions lower than that observed for the other genotypes (Table 2). Gepotidacin’s MIC_90_ value (1 µg/mL) for isolates with ParC D86N mutations was generally consistent with that of the overall population (0.5 µg/mL) with 91.5% of isolates with ParC D86N mutations susceptible to gepotidacin. Overall, the percentage of isolates for all drug-resistant genotypic subgroups that were susceptible or intermediate to gepotidacin ranged from 90% to 100% and 0% to 10%, respectively (Table 2).

Among rectal *N. gonorrhoeae* isolates with QRDR and Mtr mutations, a gepotidacin MIC_90_ of 0.5 µg/mL was observed across all genotypic subcategories, except for isolates harboring ParC S87R mutations or the GyrA S91F, D95A, and ParC S87R mutation combination, for which the gepotidacin MIC_90_ was 0.25 µg/mL (Table S1). For pharyngeal isolates, a gepotidacin MIC_90_ of 1 µg/mL was observed across QRDR and Mtr mutation subgroups, while gepotidacin MIC_90_ values for isolates with ParC S87R mutations or the GyrA S91F, D95A, and ParC S87R mutation combination were 0.25 µg/mL (Table S2). Isolates in all drug-resistant genotypes were susceptible to gepotidacin at both the rectal and pharyngeal sites (Tables S1 and S2).

#### MLST and NG-MAST epidemiological clones

Gepotidacin activity was also evaluated against the MLST and NG-MAST epidemiological clones present in urogenital *N. gonorrhoeae* isolates obtained from the micro-ITT population (Table S3). Epidemiological clone categories with *n* ≥ 10 isolates were analyzed, and the number of isolates recovered for the various clones was small, ranging from 12 to 34 for MLST clones and 10 to 21 for NG-MAST clones. The gepotidacin MIC_90_ value for MLST and NG-MAST clones was ≤1 µg/mL (range 0.12–1 µg/mL). All isolates in the most prevalent MLST and NG-MAST epidemiological clones analyzed were susceptible to gepotidacin, with the exception of two MLST 9363 isolates that had gepotidacin MICs of 2 µg/mL (intermediate susceptibility).

Gepotidacin activity against MLST and NG-MAST epidemiological clones in the micro-ITT rectal and micro-ITT pharyngeal populations is not presented due to the low number of isolates.

#### Sex/sexual orientation and country

Gepotidacin activity against urogenital *N. gonorrhoeae* isolates by sex/sexual orientation subgroups and country is provided in Table 3. Gepotidacin MIC_90_s were similar between the sex/sexual orientation subgroups, ranging from 0.5 to 1 µg/mL.

**TABLE 3:** Gepotidacin MIC frequency distribution and summary statistics for urogenital *N. gonorrhoeae* by sex/sexual orientation and country at baseline (micro-ITT population)^*a*^

Subgroup	No. of isolates	No. of isolates (cumulative %) inhibited with gepotidacin MIC (µg/mL)^*b*^						MIC_50_ (µg/mL)^*c*^	MIC_90_ (µg/mL)^*d*^	Interpretation (*n* [%])^*e*^		
		≤0.06	0.12	0.25	0.5	1	2			Susceptible	Intermediate	Resistant
All isolates	408	40 (9.8)	98 (33.8)	170 (75.5)	70 (92.6)	26 (99.0)	4 (100)	0.25	0.5	404 (99.0)	4 (1.0)	0
Sex/sexual orientation												
MSM^*f*^	290	28 (9.7)	67 (32.8)	122 (74.8)	53 (93.1)	18 (99.3)	2 (100)	0.25	0.5	288 (99.3)	2 (0.7)	0
MSW	82	7 (8.5)	20 (32.9)	35 (75.6)	15 (93.9)	3 (97.6)	2 (100)	0.25	0.5	80 (97.6)	2 (2.4)	0
Female	36	5 (13.9)	11 (44.4)	13 (80.6)	2 (86.1)	5 (100)	0 (100)	0.25	1	36 (100)	0	0
Country												
United States	53	4 (7.5)	11 (28.3)	23 (71.7)	12 (94.3)	3 (100)	0 (100)	0.25	0.5	53 (100)	0	0
Mexico	2	0	0	1 (50.0)	0 (50.0)	1 (100)	0 (100)	NC	NC	2 (100)	0	0
Germany	77	8 (10.4)	15 (29.9)	39 (80.5)	13 (97.4)	2 (100)	0 (100)	0.25	0.5	77 (100)	0	0
Spain	126	12 (9.5)	36 (38.1)	44 (73.0)	29 (96.0)	5 (100)	0 (100)	0.25	0.5	126 (100)	0	0
United Kingdom	78	6 (7.7)	21 (34.6)	36 (80.8)	9 (92.3)	4 (97.4)	2 (100)	0.25	0.5	76 (97.4)	2 (2.6)	0
Australia	72	10 (13.9)	15 (34.7)	27 (72.2)	7 (81.9)	11 (97.2)	2 (100)	0.25	1	70 (97.2)	2 (2.8)	0

^*a*^ Percentages are calculated from *n/N* where *N *is the total number of *N. gonorrhoeae* isolates within the subgroup. FDA, Food and Drug Administration; MIC, minimum inhibitory concentration; micro-ITT, microbiological intent-to-treat; MSM, men who have sex with men; MSW, men who have sex with women; NC, not calculated; *N. gonorrhoeae*, *Neisseria gonorrhoeae.*

^*b*^ The percentage of isolates shown for each gepotidacin MIC concentration was calculated as a cumulative value in a *post hoc* analysis. Gepotidacin MIC values ranged from ≤0.06 to 2 µg/mL; higher MIC categories are thus not shown.

^*c*^ MIC_50_ represents the minimum concentration at which 50% of tested bacterial isolates are inhibited in growth.

^*d*^ MIC_90_ represents the minimum concentration at which 90% of tested bacterial isolates are inhibited in growth.

^*e*^ Gepotidacin interpretations are based on FDA susceptibility test interpretive criteria (susceptible ≤1 µg/mL; intermediate = 2 µg/mL; and resistant ≥4 µg/mL).

^*f*^ Included bisexual male participants.

Across subgroups by country, gepotidacin MIC_90_s were similar, ranging from 0.5 to 1 µg/mL, except for Mexico (not calculated due to small number of isolates).

Susceptibility to gepotidacin was similar and ranged from 97.6% to 100% across sex/sexual orientation subgroups and from 97.2% to 100% across countries. As noted above, no gepotidacin resistant isolates were identified in this study (Table 3).

## DISCUSSION

Using the contemporary collection of gonococcal isolates from the EAGLE-1 clinical trial in uncomplicated urogenital gonorrhea (*N* = 408), we evaluated the *in vitro* activity of gepotidacin against *N. gonorrhoeae* with various drug-resistant/nonsusceptibility phenotypes and genotypes. These analyses were conducted using isolates from the EAGLE-1 primary analysis population (micro-ITT).

As previously reported in the EAGLE-1 primary analysis, this population was characterized by resistance to ciprofloxacin (54%), tetracycline (25%), and penicillin (17%), frequent QRDR mutations (59% of isolates), and measurable geographic and demographic variation. No isolates were nonsusceptible to cefixime, ceftriaxone, or resistant to spectinomycin (26).

In the present analysis, gepotidacin demonstrated overall MIC_50_ and MIC_90_ values of 0.25 and 0.5 µg/mL, respectively, with an observed MIC range of ≤0.06 to 2 µg/mL. Interpretation of these *in vitro* findings is supported by current FDA STIC for gepotidacin, which defines *N. gonorrhoeae* isolates with MICs of ≤1 µg/mL as susceptible, 2 µg/mL as intermediate, and ≥4 µg/mL as resistant (31). These criteria are based on a gepotidacin dosing regimen of 3,000 mg followed by a second 3,000 mg dose approximately 10–12 h later, consistent with the dosing regimen evaluated in EAGLE-1 (26). Applying these criteria to the urogenital isolates in the present analysis, 99% (404/408) were susceptible to gepotidacin, with 1% of isolates (4/408) classed as intermediate. Importantly, no urogenital (or any other body site) isolates were found to be resistant to gepotidacin.

To determine whether established resistance backgrounds influenced gepotidacin activity, isolates were stratified by resistance phenotype and genotype. Across all phenotypic and genotypic subgroups evaluated, gepotidacin MIC_50_ and MIC_90_ values were generally within ±1 dilution of those for the overall population, with no isolates in any phenotypic or genotypic subgroup resistant to gepotidacin based on the FDA STIC breakpoint. High percentages of gepotidacin susceptibility were observed among isolates resistant or nonsusceptible to azithromycin, ciprofloxacin, penicillin, tetracycline, and multidrug-resistant isolates (97.3–98.6%) and a similar trend was observed across isolates harboring QRDR mutations, including GyrA and ParC mutations (91.5–100%). Isolates harboring ParC D86N mutations showed a gepotidacin MIC_90_ value consistent with the overall population; however, a slightly higher proportion were categorized as intermediate (8.5%, four isolates) to gepotidacin compared with other QRDR genotypes (0–4.9%). Regardless, this preserved activity suggests that QRDR mutations, including ParC D86N and mutations commonly associated with fluoroquinolone resistance (GyrA S91F, GyrA D95A, and ParC S87R), did not substantially reduce gepotidacin susceptibility (91.5–100%), aligning with gepotidacin’s distinct binding profile and well-balanced dual-enzyme inhibition (18, 20, 32). In addition, gepotidacin susceptibility was ≥90% against isolates with mtrR and 23S rRNA mutations.

The four isolates with a gepotidacin MIC of 2 µg/mL were located at the upper end of the unimodal wild-type MIC distribution and fell within the expected variability of MIC testing (±1 doubling dilution). No unique resistance mechanism was identified in these isolates, and they exhibited phenotypic and genotypic profiles similar to those of isolates with lower gepotidacin MICs. Collectively, these findings suggest that the observed MIC values most likely reflect normal MIC testing variability rather than reduced gepotidacin susceptibility. Overall, activity and susceptibility were maintained, with ≥90% of isolates with drug-resistant phenotypes or genotypes susceptible to gepotidacin. Collectively, these findings indicate that common resistance mechanisms which limit activity in other antimicrobial classes were not associated with meaningful reductions in gepotidacin *in vitro* activity against *N. gonorrhoeae* urogenital isolates.

Across the most prevalent MLST and NG-MAST types evaluated, gepotidacin susceptibility was 100% with the exception of MLST 9363, which was 91%. As gonococcal resistance often spreads through clonal expansion of successful lineages (33), this uniform activity against epidemiological clones indicates that circulating strain backgrounds in this cohort were generally not associated with reduced gepotidacin susceptibility. Susceptibility was likewise consistent across sex/sexual orientation (97.6–100%) and geographic subgroups (97.2–100%), including MSM populations and countries with historically elevated resistance rates, indicating preserved activity across diverse epidemiologic settings.

Consistent with the urogenital findings, comparable *in vitro* activity and susceptibility were observed among rectal and pharyngeal *N. gonorrhoeae* isolates. All extragenital isolates were classified as susceptible to gepotidacin according to FDA STIC criteria, irrespective of drug-resistant phenotype or genotype (31). Gepotidacin MIC_90_ values were similar for rectal and pharyngeal isolates, generally within ±1 dilution; however, the numbers of isolates were small. These findings indicate consistent *in vitro* activity of gepotidacin across anatomical sites commonly involved in gonococcal infection.

Clinically, the consistent *in vitro* activity observed in urogenital isolates, together with consistent findings in extragenital sites, could translate into sustained efficacy of gepotidacin even in settings where first-line treatments are compromised by antimicrobial resistance mutations. Previous *in vitro* studies have demonstrated a low frequency of spontaneous resistance to gepotidacin in *N. gonorrhoeae* (24, 34). Nevertheless, these findings should be further informed by ongoing surveillance across diverse geographic regions and real-world clinical evidence to better understand the durability of this activity.

### Limitations

The analyses presented have several limitations, reflecting those of the main trial. First, the study was primarily conducted in Europe, with approximately 70% of the isolates included in the micro-ITT population being collected from participants in Germany, Spain, and the United Kingdom (UK). Participants were also predominantly White (74%) and male (92%). These factors may reduce the generalizability of the results and call for the evaluation of gepotidacin efficacy in populations that were underrepresented in the EAGLE-1 trial. Second, the number of available isolates for analysis was small for the rectal and pharyngeal body sites. This reflects the design of the trial—collection of these samples was optional rather than mandatory to reduce the burden on participants and mitigate discomfort. This resulted in few isolates available for comparative analysis from these body sites. Third, there were no isolates nonsusceptible to ceftriaxone in the trial, limiting the collection of gepotidacin *in vitro* data for such isolates. We would not anticipate ceftriaxone nonsusceptibility to impact the *in vitro* activity of gepotidacin based on the different mechanisms of action, and gepotidacin has previously been shown to maintain *in vitro* activity against a challenging collection of ceftriaxone-nonsusceptible isolates (24, 25, 35). Further studies are required, however, to confirm this hypothesis.

### Conclusion

In conclusion, gepotidacin was active against the *N. gonorrhoeae* isolates recovered in the EAGLE-1 phase III clinical trial. Overall, 99% of urogenital isolates were susceptible to gepotidacin at the FDA STIC breakpoint of ≤1 µg/mL, and activity was maintained against drug-resistant phenotypes and genotypes and isolates from different participant subgroups.

These data support the potential for gepotidacin as an oral, first-in-class antibacterial for the treatment of uncomplicated urogenital gonorrhea caused by a broad range of *N. gonorrhoeae* strains, complementing the primary efficacy outcomes of EAGLE-1 (26).

## Data Availability

Anonymized individual participant data and study documents (including the protocols and statistical analysis plans) can be requested for further research from https://www.gsk-studyregister.com/en/.

## SUPPLEMENTAL MATERIAL

Supplemental material aac.00752-26-s0001.docx Tables S1 to S3.

ASM does not own the copyrights to Supplemental Material that may be linked to, or accessed through, an article. The authors have granted ASM a non-exclusive, world-wide license to publish the Supplemental Material files. Please contact the corresponding author directly for reuse.
