# Supplementary material for: In vitro activity of gepotidacin against baseline Neisseria gonorrhoeae phenotypes and genotypes recovered from a clinical trial for uncomplicated urogenital gonorrhea (EAGLE-1)
Source: Antimicrob Agents Chemother. 2026 Oct 7;70(11):e00752-26. doi: 10.1128/aac.00752-26 (PMC13731185; doi:10.1128/aac.00752-26)
Supplement: Supplementary file 1 [file aac.00752-26-s0001.docx]

**Supplementary Material**

**TABLE S1.** Gepotidacin MIC frequency distribution and summary statistics against rectal *N. gonorrhoeae* isolates by phenotype and genotype at baseline (*N* = 41 isolates) (micro-ITT rectal population).

| **Phenotype or genotype subgroup^a^** | **No. of isolates** | **No. of isolates (cumulative %) inhibited with gepotidacin MIC (µg/mL):** | | | | | | **MIC_50_, µg/mL^b^** | **MIC_90_, µg/mL^c^** | **Interpretation (*n* [%])^d^** | | |
| --- | --- | --- | --- | --- | --- | --- | --- | --- | --- | --- | --- | --- |
|  |  | **≤0.06** | **0.12** | **0.25** | **0.5** | **1** | **2** |  |  | **Susceptible** | **Intermediate** | **Resistant** |
| ***All isolates*** | 41 | 5  (12.2) | 13 (43.9) | 12 (73.2) | 10 (97.6) | 1  (100) | 0 (100) | 0.25 | 0.5 | 41  (100) | 0 | 0 |
| ***Phenotypes, n (%)*** | | | | | | | | | | | | |
| Ciprofloxacin-resistant | 23 | 4  (17.4) | 7  (47.8) | 6  (73.9) | 5  (95.7) | 1  (100) | 0 (100) | 0.25 | 0.5 | 23  (100) | 0 | 0 |
| ***Genotypes, n (%)*** | | | | | | | | | | | | |
| QRDR mutation | 25 | 4  (16.0) | 9  (52.0) | 6  (76.0) | 5  (96.0) | 1  (100) | 0 (100) | 0.12 | 0.5 | 25  (100) | 0 | 0 |
| GyrA mutation | 25 | 4  (16.0) | 9  (52.0) | 6  (76.0) | 5  (96.0) | 1  (100) | 0 (100) | 0.12 | 0.5 | 25  (100) | 0 | 0 |
| GyrA S91F, D95A | 23 | 4  (17.4) | 7  (47.8) | 6  (73.9) | 5  (95.7) | 1  (100) | 0 (100) | 0.25 | 0.5 | 23  (100) | 0 | 0 |
| ParC mutation | 21 | 4  (19.0) | 7  (52.4) | 5  (76.2) | 4  (95.2) | 1  (100) | 0 (100) | 0.12 | 0.5 | 21  (100) | 0 | 0 |
| ParC S87R mutation | 16 | 3  (18.8) | 7  (62.5) | 5  (93.8) | 1  (100) | 0 (100) | 0 (100) | 0.12 | 0.25 | 16  (100) | 0 | 0 |
| GyrA S91F, D95A and ParC S87R mutations | 15 | 3  (20.0) | 6  (60.0) | 5  (93.3) | 1  (100) | 0 (100) | 0 (100) | 0.12 | 0.25 | 15  (100) | 0 | 0 |
| MtrCDE promoter mutation | 12 | 1  (8.3) | 1  (16.7) | 3  (41.7) | 7  (100) | 0 (100) | 0 (100) | 0.5 | 0.5 | 12  (100) | 0 | 0 |
| MtrC mutation | 12 | 1  (8.3) | 1  (16.7) | 3  (41.7) | 7  (100) | 0 (100) | 0 (100) | 0.5 | 0.5 | 12  (100) | 0 | 0 |
| MtrD mutation | 12 | 1  (8.3) | 1  (16.7) | 3  (41.7) | 7  (100) | 0 (100) | 0 (100) | 0.5 | 0.5 | 12  (100) | 0 | 0 |
| MtrE mutation | 12 | 1  (8.3) | 1  (16.7) | 3  (41.7) | 7  (100) | 0 (100) | 0 (100) | 0.5 | 0.5 | 12  (100) | 0 | 0 |
| MtrR coding mutation | 12 | 1  (8.3) | 1  (16.7) | 3  (41.7) | 7  (100) | 0 (100) | 0 (100) | 0.5 | 0.5 | 12  (100) | 0 | 0 |

Percentages are calculated from *n/N* where *N* is the total number of *N. gonorrhoeae* isolates within the group or subgroup.

*^a^*Phenotype and genotype categories with *n* ≥10 isolates are shown.

*^b^*MIC_50_ represents the minimum concentration at which 50% of tested bacterial isolates are inhibited in growth.

*^c^*MIC_90_ represents the minimum concentration at which 90% of tested bacterial isolates are inhibited in growth.

*^d^*Gepotidacin interpretations are based on FDA susceptibility test interpretive criteria (susceptible ≤1 µg/mL; intermediate =2 µg/mL; resistant ≥4 µg/mL).

FDA, Food and Drug Administration; MIC, minimum inhibitory concentration; micro-ITT, microbiological intent-to-treat; *N. gonorrhoeae*, *Neisseria gonorrhoeae*; QRDR, quinolone resistance-determining region.

**TABLE S2.** Gepotidacin MIC frequency distribution and summary statistics against pharyngeal *N. gonorrhoeae* isolates by phenotype and genotype at baseline (*N* = 35 isolates) (micro-ITT pharyngeal population).

| **Phenotype or genotype subgroup^a^** | **No. of isolates** | **No. of isolates (cumulative %) inhibited with gepotidacin MIC (µg/mL):** | | | | | | **MIC_50_, µg/mL^b^** | **MIC_90_, µg/mL^c^** | **Interpretation (*n* [%])^d^** | | |
| --- | --- | --- | --- | --- | --- | --- | --- | --- | --- | --- | --- | --- |
|  |  | **≤0.06** | **0.12** | **0.25** | **0.5** | **1** | **2** |  |  | **Susceptible** | **Intermediate** | **Resistant** |
| ***All isolates*** | 35 | 4 (11.4) | 8  (34.3) | 10 (62.9) | 8  (85.7) | 5  (100) | — | 0.25 | 1 | 35  (100) | 0 | 0 |
| ***Phenotypes, n (%)*** | | | | | | | | | | | | |
| Ciprofloxacin-resistant | 20 | 4 (20.0) | 4  (40.0) | 5  (65.0) | 3  (80.0) | 4  (100) | 0  (100) | 0.25 | 1 | 20  (100) | 0 | 0 |
| Tetracycline-resistant | 13 | 0 | 2  (15.4) | 3  (38.5) | 4  (69.2) | 4  (100) | 0  (100) | 0.5 | 1 | 13  (100) | 0 | 0 |
| ***Genotypes, n (%)*** | | | | | | | | | | | | |
| QRDR mutation | 22 | 4 (18.2) | 5  (40.9) | 6  (68.2) | 3  (81.8) | 4  (100) | 0 (100) | 0.25 | 1 | 22  (100) | 0 | 0 |
| GyrA mutation | 21 | 4 (19.0) | 4  (38.1) | 6  (66.7) | 3  (81.0) | 4  (100) | 0 (100) | 0.25 | 1 | 21  (100) | 0 | 0 |
| GyrA S91F, D95A mutations | 18 | 4 (22.2) | 4  (44.4) | 4  (66.7) | 3  (83.3) | 3  (100) | 0 (100) | 0.25 | 1 | 18  (100) | 0 | 0 |
| ParC mutation | 19 | 4 (21.1) | 5  (47.4) | 3  (63.2) | 3  (78.9) | 4  (100) | 0 (100) | 0.25 | 1 | 19  (100) | 0 | 0 |
| ParC S87R mutation | 12 | 4 (33.3) | 4  (66.7) | 3  (91.7) | 1  (100) | 0 (100) | 0 (100) | 0.12 | 0.25 | 12  (100) | 0 | 0 |
| GyrA S91F, D95A and ParC S87R mutations | 10 | 4 (40.0) | 3  (70.0) | 2  (90.0) | 1  (100) | 0 (100) | 0 (100) | 0.12 | 0.25 | 10  (100) | 0 | 0 |
| MtrCDE promoter mutation | 17 | 0 | 3  (17.6) | 4  (41.2) | 6  (76.5) | 4  (100) | 0 (100) | 0.5 | 1 | 17  (100) | 0 | 0 |
| MtrC mutation | 17 | 0 | 3  (17.6) | 4  (41.2) | 6  (76.5) | 4  (100) | 0 (100) | 0.5 | 1 | 17  (100) | 0 | 0 |
| MtrD mutation | 17 | 0 | 3  (17.6) | 4  (41.2) | 6  (76.5) | 4  (100) | 0 (100) | 0.5 | 1 | 17  (100) | 0 | 0 |
| MtrE mutation | 17 | 0 | 3  (17.6) | 4  (41.2) | 6  (76.5) | 4  (100) | 0 (100) | 0.5 | 1 | 17  (100) | 0 | 0 |
| MtrR coding | 17 | 0 | 3  (17.6) | 4  (41.2) | 6  (76.5) | 4  (100) | 0 (100) | 0.5 | 1 | 17  (100) | 0 | 0 |

Percentages are calculated from *n/N* where *N* is the total number of *N. gonorrhoeae* isolates within the group or subgroup.

*^a^*Phenotype and genotype categories with *n* ≥10 isolates are shown.

*^b^*MIC_50_ represents the minimum concentration at which 50% of tested bacterial isolates are inhibited in growth.

*^c^*MIC_90_ represents the minimum concentration at which 90% of tested bacterial isolates are inhibited in growth.

*^d^*Gepotidacin interpretations are based on FDA susceptibility test interpretive criteria (susceptible ≤1 µg/mL; intermediate =2 µg/mL; resistant ≥4 µg/mL).

FDA, Food and Drug Administration; MIC, minimum inhibitory concentration; micro-ITT, microbiological intent-to-treat; *N. gonorrhoeae*, *Neisseria gonorrhoeae*; QRDR, quinolone resistance-determining region.

**TABLE S3.** Gepotidacin MIC frequency distribution and summary statistics against urogenital *N. gonorrhoeae* isolates by MLST and NG-MAST epidemiological clones at baseline (*N* = 408 isolates) (micro-ITT population).

| **Epidemiological clone subgroup^a^** | **No. of isolates** | **No. of isolates (cumulative %) inhibited with gepotidacin MIC (µg/mL):^b^** | | | | | | **MIC_50_, µg/mL^c^** | **MIC_90_, µg/mL^d^** | **Interpretation (*n* [%])^e^** | | |
| --- | --- | --- | --- | --- | --- | --- | --- | --- | --- | --- | --- | --- |
|  |  | **≤0.06** | **0.12** | **0.25** | **0.5** | **1** | **2** |  |  | **Susceptible** | **Intermediate** | **Resistant** |
| ***All isolates*** | 408 | 40 (9.8) | 98  (33.8) | 170  (75.5) | 70  (92.6) | 26  (99.0) | 4  (100) | 0.25 | 0.5 | 404  (99.0) | 4  (1.0) | 0 |
| ***Epidemiological clone, n (%)*** | | | | | | | | | | | | |
| MLST | | | | | | | | | | | | |
| ST10314 | 15 | 6  (40.0) | 9  (100) | 0 (100) | 0 (100) | 0 (100) | 0 (100) | 0.12 | 0.12 | 15  (100) | 0 | 0 |
| ST11422 | 26 | 0 | 0 | 3  (11.5) | 20  (88.5) | 3  (100) | 0 (100) | 0.5 | 1 | 26  (100) | 0 | 0 |
| ST11706 | 22 | 1  (4.5) | 16  (77.3) | 5  (100) | 0 (100) | 0 (100) | 0 (100) | 0.12 | 0.25 | 22  (100) | 0 | 0 |
| ST13292 | 12 | 0 | 1  (8.3) | 11  (100) | 0 (100) | 0 (100) | 0 (100) | 0.25 | 0.25 | 12  (100) | 0 | 0 |
| ST1580 | 24 | 2  (8.3) | 2  (16.7) | 15  (79.2) | 5  (100) | 0 (100) | 0 (100) | 0.25 | 0.5 | 24  (100) | 0 | 0 |
| ST1583 | 20 | 0 | 1  (5.0) | 15  (80.0) | 4  (100) | 0 (100) | 0 (100) | 0.25 | 0.5 | 20  (100) | 0 | 0 |
| ST1599 | 22 | 0 | 0 | 18  (81.8) | 4  (100) | 0 (100) | 0 (100) | 0.25 | 0.5 | 22  (100) | 0 | 0 |
| ST7363 | 22 | 1  (4.5) | 2  (13.6) | 3  (27.3) | 8  (63.6) | 8  (100) | 0 (100) | 0.5 | 1 | 22  (100) | 0 | 0 |
| ST7822 | 29 | 14 (48.3) | 15  (100) | 0 (100) | 0 (100) | 0 (100) | 0 (100) | 0.12 | 0.12 | 29  (100) | 0 | 0 |
| ST8143 | 14 | 0 | 3  (21.4) | 5  (57.1) | 2  (71.4) | 4  (100) | 0 (100) | 0.25 | 1 | 14  (100) | 0 | 0 |
| ST8156 | 28 | 1  (3.6) | 4  (17.9) | 22  (96.4) | 1  (100) | 0 (100) | 0 (100) | 0.25 | 0.25 | 28  (100) | 0 | 0 |
| ST9362 | 34 | 2  (5.9) | 10  (35.3) | 22  (100) | 0 (100) | 0 (100) | 0 (100) | 0.25 | 0.25 | 34  (100) | 0 | 0 |
| ST9363 | 23 | 0 | 4  (17.4) | 8  (52.2) | 8  (87.0) | 1  (91.3) | 2  (100) | 0.25 | 1 | 21  (91.3) | 2  (8.7) | 0 |
| NG-MAST | | | | | | | | | |  |  |  |
| ST11461 | 11 | 1  (9.1) | 1  (18.2) | 8  (90.9) | 1  (100) | 0 (100) | 0 (100) | 0.25 | 0.25 | 11  (100) | 0 | 0 |
| ST15589 | 10 | 0 | 1  (10.0) | 7  (80.0) | 2  (100) | 0 (100) | 0 (100) | 0.25 | 0.5 | 10  (100) | 0 | 0 |
| ST17972 | 19 | 0 | 14  (73.7) | 5  (100) | 0 (100) | 0 (100) | 0 (100) | 0.12 | 0.25 | 19  (100) | 0 | 0 |
| ST19762 | 21 | 2  (9.5) | 6  (38.1) | 13  (100) | 0 (100) | 0 (100) | 0 (100) | 0.25 | 0.25 | 21  (100) | 0 | 0 |
| ST19792 | 13 | 3  (23.1) | 10  (100) | 0  (100) | 0 (100) | 0 (100) | 0 (100) | 0.12 | 0.12 | 13  (100) | 0 | 0 |
| ST20981 | 10 | 1  (10.0) | 1  (20.0) | 8  (100) | 0 (100) | 0 (100) | 0 (100) | 0.25 | 0.25 | 10  (100) | 0 | 0 |
| ST21218 | 11 | 2  (18.2) | 2  (36.4) | 0 (36.4) | 2  (54.5) | 5  (100) | 0 (100) | 0.5 | 1 | 11  (100) | 0 | 0 |
| ST3935 | 15 | 0 | 0 | 0 | 12  (80.0) | 3  (100) | 0 (100) | 0.5 | 1 | 15  (100) | 0 | 0 |

Percentages are calculated from *n/N* where *N* is the total number of *N. gonorrhoeae* isolates within the group or subgroup.

*^a^*Epidemiological clone categories with *n* ≥10 isolates are shown.

*^b^*The percentage of isolates shown for each gepotidacin MIC concentration was calculated as a cumulative value in a *post-hoc* analysis.

*^c^*MIC_50_ represents the minimum concentration at which 50% of tested bacterial isolates are inhibited in growth.

*^d^*MIC_90_ represents the minimum concentration at which 90% of tested bacterial isolates are inhibited in growth.

*^e^*Gepotidacin interpretations are based on FDA susceptibility test interpretive criteria (susceptible ≤1 µg/mL; intermediate =2 µg/mL; resistant ≥4 µg/mL).

FDA, Food and Drug Administration; MIC, minimum inhibitory concentration; micro-ITT, microbiological intent-to-treat; MLST, multi-locus sequence typing; *N. gonorrhoeae*, *Neisseria gonorrhoeae*; NG-MAST, *Neisseria gonorrhoeae* multi-antigen sequence typing.
